# Three-Year Safety and Efficacy of Endovascular Treatment of Common Femoral Artery in 150 PAD Patients

**DOI:** 10.3390/biomedicines12102213

**Published:** 2024-09-27

**Authors:** Patricia Wischmann, Manuel Stern, David-Ioan Florea, Luise Neudorf, Yassine Haddad, Nicolas Kramser, Miriam Schillings, Sven Baasen, Johanna Schremmer, Christian Heiss, Malte Kelm, Lucas Busch

**Affiliations:** 1 Division of Cardiology, Pulmonology and Vascular Medicine, Medical Faculty, Heinrich Heine University of Duesseldorf, Moorenstr. 5, 40225 Düsseldorf, Germany; manuel.stern@med.uni-duesseldorf.de (M.S.); david-ioan.florea@med.uni-duesseldorf.de (D.-I.F.); luise.neudorf@med.uni-duesseldorf.de (L.N.); yassine.haddad@med.uni-duesseldorf.de (Y.H.); nicolas.kramser@med.uni-duesseldorf.de (N.K.); miriam.schillings@med.uni-duesseldorf.de (M.S.); sven.baasen@med.uni-duesseldorf.de (S.B.); malte.kelm@med.uni-duesseldorf.de (M.K.); hanslucas.busch@med.uni-duesseldorf.de (L.B.); 2 CARID—Cardiovascular Research Institute Düsseldorf, Medical Faculty, University Hospital Düsseldorf, Heinrich-Heine-University Düsseldorf, 40225 Düsseldorf, Germany; 3 Department of Clinical and Experimental Medicine, Faculty of Health and Medical Sciences, University of Surrey, Guildford GU2 7YH, UK; c.heiss@surrey.ac.uk; 4 Department of Vascular Medicine, Surrey and Sussex NHS Healthcare Trust, Redhill RH1 5RH, UK

**Keywords:** common femoral artery, endovascular therapy, directional atherectomy

## Abstract

**Background:** The gold standard treatment for peripheral arterial disease (PAD) of the common femoral artery (CFA) is open common femoral endarterectomy (CFAE). Interest in the less invasive endovascular treatment (EVT) is growing due to PAD patients’ frequent co-morbidities. **Aims:** We aimed to evaluate three-year EVT outcomes in multimorbid PAD patients with severe calcified CFA lesions. **Methods:** Using the prospectively maintained “all-comers” Duesseldorf PTA Registry, we analysed the three-year outcomes of 150 patients with EVT of the CFA. Between January 2017 and October 2023, 66 patients received a rotational excisional atherectomy (REA) followed by a drug-coated balloon angioplasty (DCB), and 84 patients received a DCB alone. **Results:** All procedures involved the CFA, 49% additionally involved the proximal superficial femoral artery (SFA), and 10% of the lesions involved the profunda femoris artery (PFA). The procedural success rate was 97% and independent of PAD stage, with a higher level of stent implantation in the DCB group (58% vs. 39%, *p* < 0.05). The primary patency rate at one year was 83% for REA + DCB and 87% for DCB (*p* = 0.576), while secondary patency after three years was 97%. The MALE rate at three years was mainly driven by cdTLR (REA + DCB: (20%) vs. DCB: (14%), *p* = 0.377), while major amputations were low in both groups (REA + DCB: 3% vs. DCB: 1%). Overall, the major adverse cardiovascular events (MACEs) rate at three years was low (REA + DCB: (5%) vs. DCB: (11%), *p* = 0.170). **Conclusions:** The EVT of severely calcified CFA lesions is safe and effective, with high three-year patency rates and low rates of major adverse limb events (MALEs) and MACEs. This registry demonstrates that vessel preparation with REA minimizes the need for stenting.

## 1. Introduction

Common femoral artery (CFA) disease can cause claudication and chronic limb-threatening ischemia (CLTI) and is often part of broader atherosclerosis affecting the aortoiliac or femoropopliteal regions. A common femoral endarterectomy (CFEA) is the traditional primary treatment for CFA lesions and is recommended by current guidelines [1,2]. Percutaneous intervention is usually preferred for revascularizing lower limb atherosclerosis, but CFA bifurcation stenosis is often managed surgically due to its accessibility and the favourable long-term outcomes of endarterectomy [3,4]. Patients with peripheral artery disease (PAD) often have co-morbidities. They are at a high risk of surgical complications and are associated with morbidity and mortality [5,6]. Recent advances in endovascular therapy and operator expertise have increased the use of percutaneous interventions for the CFA, which now show a high rate of immediate technical success [7,8,9,10]. There are limited data on long-term endovascular treatment (EVT) outcomes for CFA bifurcation stenoses, including superficial femoral artery (SFA) and profunda femoral artery (PFA) lesions. Severe calcifications often lead to suboptimal balloon dilation results due to vessel recoil, dissections, and the need for bail-out stenting, which may jeopardise the PFA ostium. Vessel preparation with rotational excisional atherectomy (REA) improves acute success rates and primary patency by reducing the fibrocalcific component of atherosclerotic plaques [11]. Several clinical trials indicate that an atherectomy may result in lower rates of bailout stenting when compared with balloon angioplasty [12,13]; although, it is important to note that the risk of macroembolization associated with atherectomy is substantially higher [12].

This large-scale study evaluates the safety, procedural success, and one- to three-year patency of EVT for the CFA, comparing primary vessel preparation with an REA and primary treatment with a DCB.

### 1.1. Patients and Methods

We prospectively enrolled 150 patients from January 2017 to October 2023 for elective or emergency EVT in the Düsseldorf PTA Registry (NCT02728479). CFA lesions were treated with REA + DCB or DCB alone based on the interventional physician’s discretion. The study followed local ethics committee approval and the Declaration of Helsinki. Baseline characteristics, including cardiovascular comorbidities, peripheral artery type, and vital signs, were recorded. Chronic kidney disease (CKD) is classified into stages based on kidney function and damage: Stage I has normal or slightly increased kidney function with evidence of damage (GFR ≥ 90 mL/min/1.73 m^2^), Stage II has mild reduction in function with damage (GFR 60–89 mL/min/1.73 m^2^), and Stage III has moderate reduction in function (GFR 30–59 mL/min/1.73 m^2^). Clinical status was assessed using the Fontaine classifications. Periprocedural risks were evaluated using the National Cardiovascular Data Registry (NCDR) bleeding risk and NCDR mortality risk. Admission blood parameters and medical history, including antiplatelet and anticoagulant treatments, were documented.

The anatomic inclusion criteria were:

- Calcified lesion located at the CFA, proximal SFA, and proximal PFA
- Diameter stenosis >70%
- Vessel diameter of 5 to 7 mm

The anatomic exclusion criteria were:

- In-stent restenosis or thrombosis
- Distal SFA lesions and distal PFA
- Diameter stenosis <70%

Endovascular treatment included the combined use of REA (Rotarex™ Rotational Excisional Atherectomy System, BD^®^, Franklin Lakes, NJ, USA) followed by a DCB treatment (Passeo^®^-18 Lux^®^; Biotronik^®^, Berlin, Germany or Ranger ^®^; Boston Scientific^®^, Marlborough, MA, USA) or the use of DCB alone.

Visual calcium scoring was performed across different femoral segments using duplex sonography. The duplex scan provided detailed information on the degree of stenosis by assessing blood flow velocities and vessel diameter. A score of 0 was assigned if no wall heterogeneity or anechoic shadowing was observed. A score of 1 was given when wall heterogeneity was present without anechoic shadowing (diameter stenosis < 70%). A score of 2 was assigned if clear anechoic shadowing was observed or in cases of high-grade stenosis (diameter stenosis > 70%) or total occlusion.

Bifurcation lesions were classified according to the Medina classification. This classification was developed for the coronary arteries and recently also used for the description of femoral bifurcation lesions. For the characterization of femoral bifurcation lesions, the Medina classification was defined as follows: 1-0-0 is as a single CFA lesion, 1-1-0 is a CFA and SFA lesion, 1-0-1 is defined as a CFA and PFA lesion, and 1-1-1 is used for calcification of all three vessels (CFA + SFA + PFA). CFA lesions can vary in terms of location (exclusive CFA involvement vs. extension into the femoral bifurcation or external iliac artery) and morphology (e.g., degree of calcification). Thus, a standardized classification system is needed to facilitate the comparison of published data and to identify treatment strategies that best match individual lesion characteristics. Thus, the modified coronary Medina classification proposed by Bonvini et al. is limited by considering only the anatomical lesion location and lesion extension [14].

### 1.2. Interventional Technique

All procedures were performed percutaneously, with the patient under local anaesthesia. Given the typically complex nature of CFA bifurcation stenosis or occlusions, these were treated in single sessions. Vascular access was achieved via the contralateral common femoral artery. Therefore, we inserted a 45 cm long 6 or 8 Fr sheath (Fortress, Biotronik^®^; Berlin, Germany). Once diagnostic angiography was completed, a wire (0.018”), chosen by the operator according to the stenosis type, was navigated into the distal SFA. In the case of total occlusion, a 0.018” or 0.035” wire was used to cross the lesion, if necessary, with a Navicross^®^ 18 or 35 (Terumo, Tokyo, Japan) support catheter. To allow lesion crossing with a DCB or REA device, lesion preparation was performed with an undersized balloon. In the case of an REA, we used the Rotarex TM 6F or 8F system. When the PFA or the SFA was affected, an REA was performed in each of them. When angiograms demonstrated that residual stenosis was lower than 30%, a post-dilation with a DCB (sizing was 1:1 to the reference vessel diameter and 10 mm longer than the stenosis) for at least 120 s was performed. The DCB devices used were the Passeo^®^-18 Lux^®^ (Biotronik^®^) or Ranger^®^ (Boston Scientific^®^). In the DCB alone group, recanalization was primarily performed, as described above. Provisional stenting was applied in both groups in the event of a suboptimal result, i.e., flow-limiting dissection, acute vessel occlusion, or residual stenosis >50%. In all cases, we selected self-expanding nitinol stents with a slightly oversized diameter (i.e., 1.1:1 ratio). Procedural time was defined as the time from the completion of diagnostic angiography to the final views. Technical success was defined as the absence of significant residual stenosis as less than 30% residual stenosis after EVT. 

### 1.3. Post-Procedure Patient Management

In most cases, a vascular closure device (Angio-Seal™ VIP, Terumo^®^) was placed. Patients were then immobilized in the regular ward for 4 h with monitoring of foot pulses and clinical puncture site. Post-procedural follow-up with duplex sonography, ankle brachial index (ABI) measurement, and pulse oscillography was performed the following day. A complete blood count was obtained post-procedure. Antithrombotic therapy, typically dual antiplatelet therapy (aspirin (ASA) + clopidogrel) for one month, was at the interventionalist’s discretion, followed by lifelong ASA or rivaroxaban 2.5 mg twice daily with ASA (COMPASS/VOYAGER) [15,16]. In cases where dual antiplatelet therapy or effective oral anticoagulation was already established, it was continued [17].

### 1.4. Patient Follow-Up

Patients were evaluated up to hospital discharge at 3, 12, 24, and 36 months post-procedure. The follow-up visits at each interval included physical and clinical examination, assignment of a Rutherford classification, arterial Doppler occlusion pressure measurements with the calculation of the ABI, and colour duplex sonography. Calculation of the ABI ratios was performed by recording the patient’s brachial systolic pressure, posterior tibial artery systolic pressure, and dorsalis pedis artery systolic pressure on both sides of the body. A complete blood count was obtained after the procedure. 

### 1.5. Endpoint Definitions

This analysis aimed to investigate the technical success rate, safety, and long-term outcomes of the EVT of CFA bifurcation stenosis. Safety was assessed based on in-hospital major and minor complications. The major complications included major bleeding (defined by haemoglobin (Hb) drop > 3 g/dL from baseline Hb level) and the need to convert to surgery, while minor complications were defined as embolism and minor bleeding (defined by Hb drop < 3 g/dL from baseline Hb level). Long-term outcomes included clinical-driven target lesion revascularization (cdTLR), primary patency, secondary patency, major adverse limb events (MALEs), and major adverse cardiovascular events (MACEs). CdTLR was defined as any revascularization procedure (such as angioplasty, stenting, or bypass surgery) performed due to the recurrence of symptoms or clinical evidence of ischemia at the site of a previously treated lesion. Primary patency was defined as the absence of restenosis (restenosis < 30%) in the treated vessel segment, as evaluated by duplex sonography. Secondary patency was defined as freedom from symptoms and revascularization after a re-intervention (e.g., thrombectomy, thrombolysis, angioplasty) had taken place to restore patency. MALEs included cdTLR and unplanned major limb amputation, while MACEs encompassed heart failure, non-fatal stroke, non-fatal myocardial infarction, and cardiovascular death during a three-year follow-up.

### 1.6. Statistical Analysis

Continuous variables were reported as means with standard deviations, and categorical variables as frequencies and percentages. Normally distributed continuous variables were analysed with an independent t-test, non-normally distributed with the Mann–Whitney U test, and categorical variables with chi-squared analysis. Data were processed using IBM SPSS Statistics Version 23.0. Normality was tested with the Shapiro–Wilk test, and a *p*-value < 0.05 was considered significant. Adverse event rates were estimated with Kaplan–Meier analysis, and differences between REA + DCB and DCB were assessed with the log-rank (Mantel–Cox) test. Cox regression, adjusted for age, sex (male), diabetes mellitus, (DM) body mass index (BMI), estimated glomerular filtration rate (GFR), CLTI, IC, and stent placement, was used to account for confounding variables.

## 2. Results

### 2.1. Patient Characteristics

The baseline clinical characteristics of patients with REA + DCB and DCB alone are shown in (Table 1). The incidence of CLTI was higher in the DCB group when compared with the REA + DCB group (*p* = 0.317). Patients with a DCB were characterized by an increased number of smokers (n = 34 (40%) vs. n = 15 (23%); *p* = 0.038); whereas, other cardiovascular risk factors did not differ between both groups. Moreover, patients in the REA + DCB group were more likely to be treated with (N)OACs (n = 19 (29%) vs. n = 19 (23%); *p* = 0.389). Baseline demographics and periprocedural risk scores were not statistically different. The ABI was in both treatment groups pre-procedural < 0.70 and did not differ between both groups (Table 2).

### 2.2. Lesion Characteristics

Out of a total of 150 cases, all cases underwent single CFA (n = 76) or combined CFA and proximal SFA (n = 58) intervention. A detailed description of the treated vessel segment is shown in Table 2 (Medina class). In 125 cases (83%), chronic subtotal occlusions were recanalized. 

In both treatment groups, a pre-procedural stenosis degree of 94.0 ± 11.9% was observed, with the proportion of stenosis significantly higher (*p* = 0.016) in the DCB alone group (96.1 ± 13.3%) compared with the REA + DCB group (91.6 ± 9.2%).

The level of calcification was high in both groups, according to visual calcium scoring by duplex sonography [18] (Table S1). 

The post-procedural stenosis degree was similar between groups (*p* = 0.652), with a technical success rate of 97% (n = 146). Both groups showed no differences in flow profiles, but biphasic flow increased significantly compared with pre-procedural measurements, accompanied by an improved ABI (Table 2).

### 2.3. Procedural Characteristics 

Both groups exhibited vascular occlusion with severe calcification (REA + DCB: n = 64 (100%) vs. DCB alone: n = 80 (95%), *p* = 0.291) (Table S1). All procedures were performed using a crossover access. Larger sheath sizes were used for the REA (Table 2). The mean procedural time was 91 ± 40 min, and the mean contrast volume was 73 ± 37 mL with a higher contrast dose in the REA + DCB group (REA + DCB: 85 ± 40 mL vs. DCB alone: 63 ± 31 mL; *p* = <0.001) (Table 2).

### 2.4. Technical Success

Technical and procedural success, defined as residual stenosis < 30% at the end of the procedure, was achieved in 97% of patients (146/150), with only minor procedure-related adverse events occurring. In the DCB group, 49 patients (58%) required stent implantation due to flow-limiting dissection (n = 21 (25%)) (Table 2). In the REA + DCB group, 3 patients (2%) needed local thrombus aspiration for below-the-knee embolization (Table 3).

The safety results after EVT were promising, as almost 99% of complications occurred during the same procedure and were treated percutaneously. Procedural complications occurred in 6 of 150 patients (4%), with 1% being minor complications and 3% being major complications. Minor complications were mostly driven by peripheral embolism and minor bleeding (defined by Hb drop < 3 g/dL from baseline Hb level). Major complications were rare overall, but they occurred exclusively in the DRA group. During our study, major complications occurred in 4 patients in the REA + DCB group (3%). In 1 of 4 patients an endovascular endoprosthesis was required due to significant bleeding (defined as a drop in Hb ≥ 3 g/dL compared with baseline levels). This procedure was performed without closing the PFA ostium (Table 3). In the REA + DCB group, more intra-procedural bleedings occurred (REA + DCB: n = 3 (5%) vs. DCB alone: n = 0 (0%); *p* = 0.048).

### 2.5. Clinical and Procedural Outcomes during a Three-Year Follow-Up Period after Endovascular Treatment

Table 4 reports clinical and procedural outcomes as well as target vessel revascularization (TVR) and cdTLR rates, at three-year follow-up. Duplex ultrasound or clinical follow-up controls were available in 138 of 150 patients (92%) for a follow-up of three years. 

After the initial revascularization of the target lesion vessel, the one-year patency rate was 85% (n = 128), which remained at 83% (n = 125) at three years, demonstrating the efficacy of EVT. Patients with symptomatic restenosis confirmed by duplex ultrasound underwent cdTLR during follow-up, achieving a three-year secondary patency rate of 97% (n = 146), with no significant differences between intervention groups (*p* = 0.763).

At 3 years, the mean ABI was significantly higher than the baseline in both groups (*p* < 0.05).

The cdTLR rate at 36 months was 17% (25/150 patients), with the primary endpoint (freedom from cdTLR) achieved at 85% (n = 128). Most cdTLR cases (18/25) were treated percutaneously or conservatively (Table 4).

There was a trend towards prolonged wound healing in the DCB alone group compared with the REA + DCB group (REA + DCB: 95 ± 26 days vs. DCB alone: 120 ± 32 days; *p* = 0.071). 

Major limb amputation was rare at three years (REA + DCB: n = 2 (3%) vs. DCB alone: n = 1 (1%), *p* = 0.493), as was minor limb amputation (REA + DCB: n = 0 (0%) vs. DCB alone: n = 1 (1%), *p* = 0.869). MACEs were documented in 12 out of 150 patients (7%) at three years with a higher incidence in the DCB alone group as compared with the REA + DCB group (REA + DCB: n = 3 (5%) vs. DCB alone: n = 9 (11%), *p* = 0.170). The all-cause mortality rate after three years was higher in the DCB alone cohort (REA + DCB: n = 3 (5%) vs. DCB alone: n = 12 (14%), *p* = 0.074), primarily due to cardiovascular events.

### 2.6. Clinical Outcome Prediction after Endovascular Treatment

**Figure 1:**
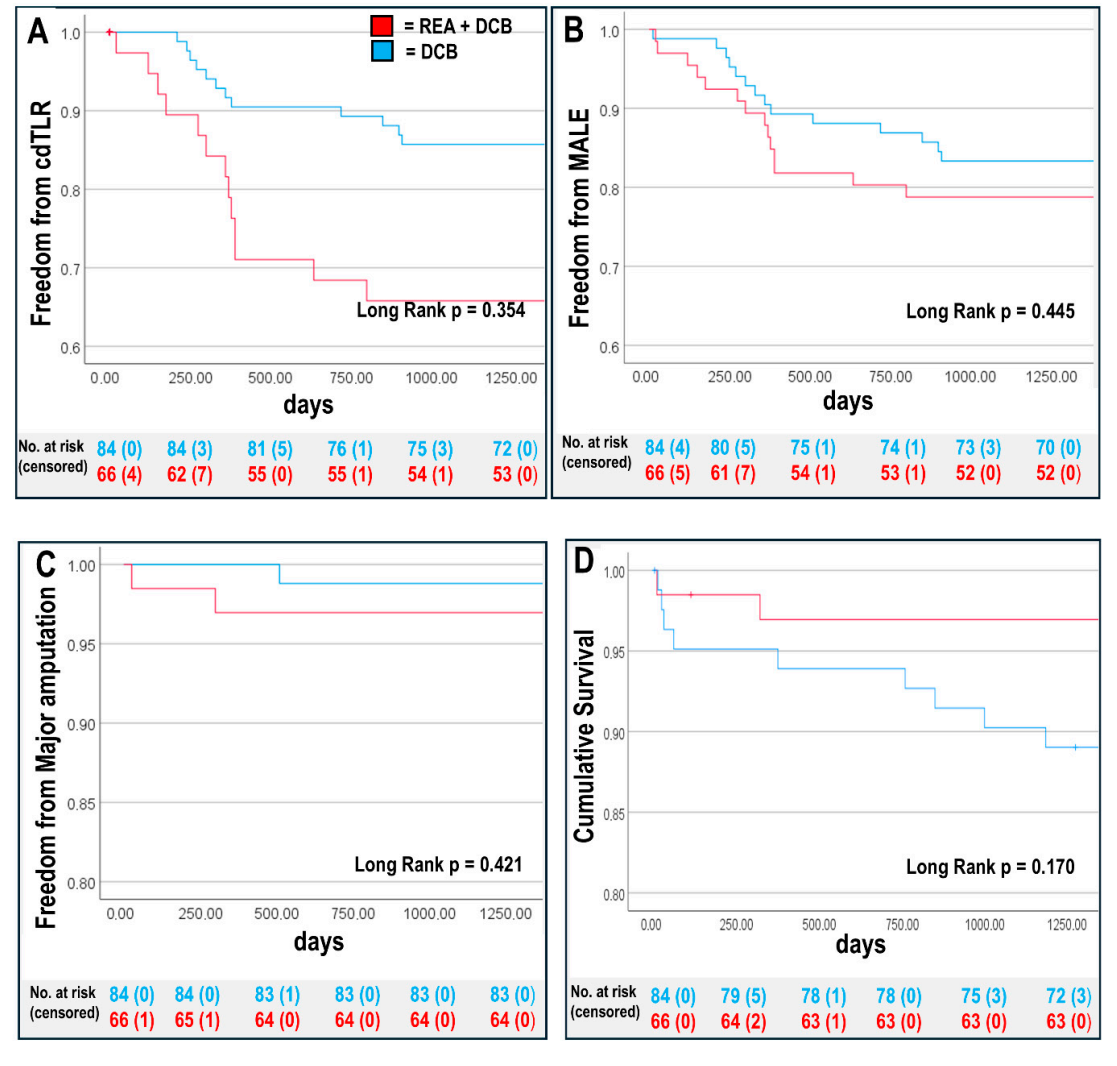
Kaplan–Meier survival analysis at three years for REA + DCB group and DCB group (**A**) cdTLR-free survival rate, (**B**) freedom from major adverse limb event (MALE: major amputation and clinical-driven target lesion revascularisation [cdTLR]), (**C**) amputation-free survival, and (**D**) overall survival rate. Overall *p*-values are shown, see Table 5 for 95% confidence intervals of individual groups.

We performed Kaplan–Meier (KM) survival analysis and compared outcomes of patients with REA + DCB (n = 66) with those that were treated with DCB alone (n = 84). Patients with REA + DCB had a lower three-year mortality (REA + DCB: n = 3 (5%) vs. DCB alone: n = 9 (11%), *p* = 0.170) (Figure 1).

We compared clinical outcomes such as cdTLR and MALEs between the EVT groups and found no differences. Patients treated with DCB alone had higher CLTI rates, linked to longer wound healing. Cox regression analysis was used to examine the association between REA + DCB and clinical outcomes adjusted for confounders including baseline age, BMI, sex (male) DM, eGFR ≤ 60 mL/min, PAD stage, and stent placement. The adjusted Cox regression analysis revealed that combining an REA with a DCB led to improved clinical outcomes for MACEs compared with using DCB alone, with other clinical endpoint parameters remaining unaffected by confounding factors. MACEs were mainly influenced by CLTI, and Kaplan–Meier survival curves largely confirmed these results (Figure 1 and Table 5).

## 3. Discussion

This large prospective study of 150 PAD patients demonstrates that endovascular treatment of heavily calcified CFA lesions is associated with high procedural success (n = 146 (97%), low complication rates (n = 4 (3%) major and n = 2 (1%) minor complications), and a high patency rate of 97%, with low MACE rates and overall low major amputation rates at three-year follow-up, highlighting the potential of EVT for limb preservation in patients with CLTI. The CdTLR and MALE rates were independent of comorbidities and PAD stage, but MACEs were predicted by CLTI. 

In addition, these data suggest that vessel preparation with an REA minimizes the need for stenting, while bailout stenting in the CFA is associated with a good three-year patency rate, highlighting EVT’s effectiveness in challenging cases. 

### 3.1. Patient Population

This study was conducted in a large cohort of high-risk patients with PAD affected by a remarkable proportion of cardiovascular risk factors. The prevalence of these comorbidities is even higher than in the recently published VOYAGER PAD trial and COMPASS trial and the previously reported German national average in hospitalized PAD patients [16,19,20]. A total of 22% of the enrolled patients are affected by CLTI, which is characterized by a high morbidity and mortality rate [6]. In addition, 75% (n = 112) of all patients had advanced kidney disease (CKD ≥ III). The study groups differed in terms of PAD stage. Patients with CLTI were more likely to be treated with DCB alone. This preference is supported by recent guidelines and studies, including the 2024 ESC guidelines, which emphasize that DCB therapy can be more appropriate for patients with advanced CLTI due to its efficacy in reducing restenosis without the added procedural complexity and discomfort associated with atherectomy. Atherectomy is characterized by longer procedure times, as in this study, which is not feasible for every CLTI patient due to pain and general discomfort. Thus, the DCB is often preferred in these cases to balance treatment efficacy with patient comfort and procedural feasibility.

### 3.2. Endovascular Treatment of Severely Calcified CFA Lesions Is Safe and Effective

The study shows that EVTs are safe and effective for multimorbid patients, with high technical success, improved ABI, and low complication rates. Endovascular studies have reported minor and major (i.e., large hematoma, haemorrhagic shock, ischemia) complications in up to 9.5% and 3.4%, respectively [14,21]. In this study, complications, including distal embolization, occurred in three REA + DCB cases. One major access site bleed occurred in the REA + DCB group during the procedure and was treated with a covered stent in the same procedure. The procedural success rate was 97% (n = 146), consistent with other studies, and was unaffected by the PAD stage or CLTI, underscoring EVT’s safety in complex cases [22]. 

### 3.3. Vessel Preparation with Directional Atherectomy Minimizes the Need for Stenting

A key finding is that stenting rates in heavily calcified CFA lesions are higher after the primary DCB compared with primary REA + DCB. Atherectomy improves lesion compliance, reducing the need for high-pressure angioplasty and dissection rates [23]. Consistent with other studies, stenting in the CFA shows low restenosis and repeat revascularization rates after one year [13,14,22]. This study indicates that a “leave-nothing-behind” approach with primary atherectomy and a DCB is effective, while stenting also maintains high patency in challenging lesions. In patients with advanced PAD, recurrent endovascular procedures are frequently performed via the femoral access. However, this approach is often not feasible if the common femoral artery has been stented. Therefore, the decision not to stent the CFA presents advantages for future endovascular procedures.

### 3.4. High Patency Rates at Three-Year Follow-Up

Primary target lesion patency rates were similar between groups at one year (n = 55 (83%) with REA + DCB vs. n = 73 (87%) with DCB, *p* = 0.576). Most patients with clinically relevant restenosis or occlusion at one year (cdTLR: REA + DCB: n = 13 (20%), DCB: n = 12 (14%), *p* = 0.377) underwent re-intervention, resulting in an overall high secondary patency rate at three years (n = 146 (97%) in both groups, *p* = 0.763), demonstrating high long-term efficacy of both EVT approaches. 

Primary target lesion patency rates vary between studies at one year. In the single-arm REA + DCB study by Cioppa et al., 6.7% restenosis (N = 2/30) and 3.3% TLR (N = 1/30) were reported in patients who underwent revascularization of severe CFA lesions with REA followed by a DCB at one-year follow-up [22]. Additionally, researchers compared REA + DCB (N = 29) with DCB alone (N = 35) for CFA occlusion, finding better outcomes with adjunctive atherectomy [13]. The one-year primary patency rates were 83% (n = 55) for the REA + DCB and 87% (n = 73) for the DCB groups, respectively, while freedom from TLR after endovascular reintervention of target lesion restenosis was 95% (n = 63) in the REA + DCB group and 96% (n = 81) in the DCB group. 

In this study, three-year patency and cdTLR were independent of PAD stage, with no increased rate of repeat procedures in CLTI patients, highlighting the efficacy of CFA EVT across PAD stages.

### 3.5. Low Rate of Major Adverse Limb Events at Three-Year Follow-Up

MALE rates were similar between REA + DCB (n = 13, 20%) and DCB alone (n = 12, 14%), driven by cdTLR, with low major amputation rates (n = 2, 3% vs. n = 1, 1%) in both groups, demonstrating EVT’s efficacy for PAD patients with calcified lesions. MALE rates were lower than other CFA intervention registries, within the 10–20% range [14,24]. Despite advances in PAD treatment, challenges such as lesion length, plaque burden, and severe calcification persist, impacting procedure performance and predicting higher restenosis rates [25,26]. Therefore, evaluating calcification severity with calcium scores is essential for EVT planning [27]. 

In recent decades, clinical studies on surgical CFA therapies have reported amputation rates ranging from 0% at 5 years [21] to 14.8% at 15 years [28]; whereas, endovascular studies report 3–6.1% major amputations after 24 months. 

### 3.6. Low Rate of MACEs at Three-Year Follow-Up

Studies show that intermittent claudication doubles the mortality rate compared with those without it, and patients with CLTI face the highest cardiovascular death risk [29]. This study confirms that individuals with CLTI and severely calcified lesions have up to twice the risk of mortality compared with those without ulceration [30]. While both treatment modalities showed similar MALE rates of cdTLR and amputation over three years, the DCB group had a higher mortality rate. These results remained consistent even after adjusting for potential confounders that could influence the clinical outcome of both EVTs. We used a DCB more commonly in patients with advanced PAD, particularly those with CLTI. Consequently, these patients have a higher mortality rate due to the advanced nature of their disease and the severity of their condition [31].

Overall, the MACE rate at three years in both groups is low compared with national registries in Germany [20]. We speculate that the low MACE rates in this study are due to: (i) thorough cardiovascular evaluations (echocardiography and ECG) at each visit; (ii) effective limb salvage, reflected in the low major amputation rate: (iii) high follow-up rates, indicating better-than-typical patient compliance [32].

### 3.7. Predictors of Outcome

Cox regression analysis was performed to assess the impact of comorbidities and PAD stage on clinical outcomes at three years. The rate of CdTLR and MALE was independent of comorbidities and PAD stage, highlighting the potential of EVT for limb preservation in patients with CLTI. The predictor of MACEs was CLTI (HR 5.0 (CI 1.002–25.366; *p* = 0.05), corroborating previous studies showing an overall high MACE rate in CLTI patients [20].

## 4. Limitations

This study is a large all-comers series with robust follow-up, providing comprehensive real-world evidence of the efficacy and safety of EVT. However, as an all-comer, prospective, single-centre study, it was not randomized. As noted in the Discussion, the PAD stage influenced the treatment arm, with the REA performed less frequently in CLTI patients due to the longer procedure time. Differences in baseline characteristics, such as disease severity, may influence outcomes and make it difficult to attribute results solely to EVT. 

## 5. Conclusions

This analysis of 150 PAD patients with CFA lesions shows that EVT with REA + DCB or DCB alone offers high success rates, low in-hospital complications, high patency, and low MALE and MACE rates at three-year follow-up, independent of the PAD stage. Vessel preparation with an REA reduces the need for stenting, while bailout stenting in the CFA still provides favourable long-term patency, highlighting the effectiveness of endovascular treatment in difficult cases.

## Figures and Tables

**Table 1:** Baseline clinical and demographical characteristics of patients with femoral bifurcation stenosis undergoing rotational excisional atherectomy (REA) and drug-coated balloon (DCB) treatment or DCB treatment alone. BMI = body mass index, CV = cardiovascular; CLTI = chronic limb-threatening ischemia; IC = intermittent claudication; CKD = chronic kidney disease; PCI = percutaneous coronary intervention; (N) STEMI = (non) ST-segment elevation myocardial infarction; ACVB = aorto-coronary venous bypass; fempop = femoropopliteal bypass, CAD = coronary artery disease; MCH = mean corpuscular haematocrit; MCV = mean corpuscular volume; MCHC = mean corpuscular haematocrit concentration; INR = international normalized ratio; CRP = C-reactive protein; eGFR = estimated glomerular filtration rate, (N)OAC = (new) oral anticoagulation; COMPASS = aspirin and low dosage of rivaroxaban t; ACE/ARB inhibitor = angiotensin converting enzyme/angiotensin receptor blocker inhibitor; SGLT2 = sodium glucose linked transporter 2; NCDR = National Cardiovascular Data Registry. Values are mean ± SD or are given as absolute values in %.

Characteristics	Femoral Bifurcation Stenosis (All)(n = 150)	REA + DCB (n = 66)	DCB Alone (n = 84)	*p*-Value REA + DCB vs. DCB Alone
Demographics				
Age (years)	72.4 ± 9.4	73.2 ± 9.3	71.7 ± 9.5	0.335
Sex (male, %)	70 (%)	67 (%)	71 (%)	0.667
BMI (kg/m^2^)	27.1 ± 4.4	27.0 ± 4.0	27.1 ± 4.6	0.825
CLTI (n, %)	33 (22%)	12 (18%)	21 (25%)	0.317
IC (n, %)	117 (78%)	54 (82%)	63 (75%)	0.317
CV risk factors				
Hypertension (n, %)	118 (79%)	52 (79%)	66 (79%)	0.974
Diabetes mellitus (n, %)	87 (58%)	37 (56%)	50 (60%)	0.670
Diabetes mellitus type 1 (n, %)	7 (5%)	4 (6%)	3 (4%)	0.656
Diabetes mellitus type 2 (n, %)	80 (53%)	33 (50%)	47 (56%)	0.656
Chronic kidney disease (n, %)				
CKD I–II (n, %)	38 (25%)	19 (29%)	19 (23%)	0.358
CKD ≥ III (n, %)	112 (75%)	47 (71%)	65 (77%)	0.568
Hyperlipidaemia (n, %)	77 (51%)	38 (58%)	39 (46%)	0.175
Smoking (n, %)	49 (33%)	15 (23%)	34 (40%)	0.038
Previous PCI (n, %)	58 (39%)	36 (55%)	22 (26%)	0.186
Previous (N) STEMI (n, %)	20 (13%)	15 (23%)	5 (6%)	0.066
Previous ACVB graft (n, %)	25 (17%)	16 (24%)	9 (11%)	0.186
Previous fempop graft (n, %)	9 (6%)	2 (3%)	7 (8%)	0.175
CAD (n, %)	98 (65%)	56 (85%)	42 (50%)	0.475
Stroke/TIA (n, %)	17 (11%)	9 (14%)	8 (10%)	0.430
Previous limb amputation (n, %)	7 (5%)	4 (6%)	3 (4%)	0.473
Laboratory parameters				
Haemoglobin (g/dL)	12.9 ± 1.9	12.8 ± 1.9	12.9 ± 1.8	0.825
Haematocrit (%)	39.5 ± 5.5	39.2 ± 5.8	39.7 ± 5.3	0.568
MCH (pg)	30.0 ± 2.5	29.8 ± 2.9	30.3 ± 2.2	0.225
MCV (fl)	92.1 ± 6.2	91.4 ± 7.6	92.6 ± 4.9	0.252
MCHC g Hb/dL	32.6 ± 1.1	32.5 ± 1.1	32.6 ± 1.0	0.713
Platelets (×1000/µL)	249.5 ± 91	245.1 ± 86	253.0 ± 93	0.597
INR	1.2 ± 0.4	1.1 ± 0.2	1.2 ± 0.5	0.101
Total cholesterol (mg/dL)	153.4 ± 41.5	150.8 ± 44.0	155.4 ± 39.5	0.521
Triglyceride (mg/dL)	139.7 ± 71.6	134.0 ± 61.7	144.4 ± 78.8	0.388
HDL (mg/dL)	49.0 ± 19.8	52.0 ± 24.7	46.7 ± 14.8	0.145
LDL (mg/dL)	85.1 ± 34.0	79.7 ± 33.5	89.3 ± 34.1	0.108
HbA1c (%)	6.9 ± 4.9	7.5 ± 6.6	6.3 ± 1.5	0.252
CRP (mg/dL)	1.4 ± 2.9	1.4 ± 2.7	1.3 ± 3.1	0.882
Creatinine (mg/dL)	1.3 ± 1.0	1.3 ± 1.1	1.4 ± 0.9	0.815
eGFR (mL/min)	62.5 ± 29.0	63.3 ± 28.3	61.8 ± 29.7	0.760
Haemodynamics				
HF (bpm)	74 ± 15	74 ± 16	75 ± 14	0.868
sBP (mmHg)	145 ± 20	141 ± 21	147 ± 19	0.081
dBP (mmHg)	81 ± 12	81 ± 11	82 ± 12	0.621
Anticoagulation				
Total (N)OAC (n, %)	38 (25%)	19 (29%)	19 (23%)	0.389
Aspirin (n, %)	6 (4%)	3 (5%)	3 (4%)	0.763
Dual anti-platelet therapy (n, %)	71 (47%)	24 (36%)	47 (56%)	0.017
COMPASS (n, %)	35 (23%)	20 (30%)	15 (18%)	0.074
Medication				
ACE/ARB inhibitor (n, %)	119 (79%)	48 (73%)	71 (85%)	0.149
Betablocker (n, %)	100 (67%)	41 (62%)	59 (70%)	0.295
Statine (n, %)	140 (93%)	63 (95%)	77 (92%)	0.356
Atorvastatine (n, %)	118 (79%)	51 (77%)	67 (80%)	0.712
Simvastatine (n, %)	22 (15%)	12 (18%)	10 (12%)	0.281
Eztimibe (n, %)	14 (9%)	10 (15%)	4 (5%)	0.030
Repatha (n, %)	2 (1%)	2 (3%)	0 (0%)	0.108
Diuretics (n, %)	48 (32%)	20 (9%)	28 (2%)	0.693
Metformin (n, %)	43 (29%)	18 (30%)	25 (33%)	0.738
Insulin (n, %)	39 (26%)	13 (20%)	26 (31%)	0.119
Sitagliptin (n, %)	11 (7%)	5 (8%)	6 (7%)	0.920
SGLT2 (n, %)	11 (7%)	9 (14%)	3 (4%)	0.024
Periprocedural risk				
NCDR bleeding risk%	4.5 ± 2.7	4.5 ± 2.2	4.5 ± 3.0	0.985
NCDR mortality risk%	1.5 ± 6.5	0.9 ± 1.1	2.0 ± 8.7	0.298

**Table 2:** Lesion characteristics, procedural details, and technical equipment of femoral bifurcation stenosis treatment. REA = rotational excisional atherectomy; DCB = paclitaxel-coated balloon; CFA = common femoral artery; SFA = superficial femoral artery; PFA = profunda femoral artery; POBA = plain old balloon angioplasty. Values are mean ± SD or are given as absolute values in %. Values are mean ± SD or are given as absolute values in %.

Procedural Characteristics	Femoral Bifurcation (All) (n = 150)	REA + DCB (n = 66)	DCB Alone (n = 84)	*p*-Value REA + DCB vs. DCB Alone
Pre-procedural degree of stenosis				
Stenosis (%)	94.0 ± 11.9	91.6 ± 9.2	96.1 ± 13.3	0.016
Post-procedural degree of stenosis				
Stenosis (%)	27.5 ± 5.3	27.3 ± 5.2	27.7 ± 5.4	0.652
Medina classification (n, %)				
1-0-0 (CFA) 1-0-1 (CFA + PFA) 1-1-0 (CFA + SFA) 1-1-1 (CFA + SFA + PFA)	76 (51%) 10 (6%) 58 (39%) 6 (4%)	28 (39%) 3 (5%) 31 (47%) 4 (6%)	48 (58%) 7 (8%) 27 (32%) 2 (2%)	0.073 0.365 0.064 0.254
ABI pre-procedural				
ABI (treated leg)	0.67 ± 0.2	0.67 ± 0.2	0.67 ± 0.2	0.450
ABI post-procedural				
ABI (treated leg)	0.77 ± 0.2	0.76 ± 0.1	0.77 ± 0.2	0.747
Access sheath size				
6 French (n, %)	112 (72%)	28 (42%)	84 (100%)	<0.001
8 French (n, %)	39 (26%)	39 (58%)	0 (0%)	<0.001
Technical data				
Number of ballons (n, %)				0.233
1 ballon	48 (32%)	31 (47%)	17 (29%)	0.233
2 ballon	74 (49%)	32 (48%)	42 (50%)	0.233
≥3 ballon	27 (18%)	17 (26%)	10 (12%)	0.233
POBA (n, %)	60 (40%)	33 (50%)	27 (32%)	0.027
Stent (n, %)	75 (50%)	26 (39%)	49 (55%)	0.048
Supera (n, %)	23 (15%)	12 (23%)	11 (13%)	0.518
Innova (n, %)	50 (33%)	12 (18%)	38 (45%)	<0.05
Viabahn (n, %)	2 (1%)	2 (3%)	0 (0%)	0.653
Procedural characteristics				
Heparine (Units)	4089 ± 1590	4309 ± 1765	3917 ± 1424	0.144
Operative time (min)	91 ± 40	97 ± 39	87 ± 40	0.130
Contrast dose (mL)	73 ± 37	85 ± 40	63 ± 31	<0.001
Flow-limiting dissection (n, %)	25 (17%)	4 (6%)	21 (25%)	<0.001
Dissection CFA	19 (13%)	3 (4%)	16 (19%)	0.008
Dissection SFA	4 (3%)	1 (2%)	3 (4%)	0.438
Dissection PFA	2 (1%)	0 (0%)	2 (2%)	0.207
Stenting (n, %)	75 (50%)	26 (39%)	49 (58%)	<0.05
Procedural Technical success				
Open vessel (n, %)	147 (98%)	63 (95%)	82 (98%)	0.464
Technical success rate (n, %)	146 (97%)	63 (95%)	81 (96%)	0.763

**Table 3:** Safety outcome of EVT of femoral bifurcation stenosis treatment. REA = rotational excisional atherectomy; DCB = paclitaxel-coated balloon. Values are mean ±  SD or are given as absolute values in %.

Safety Characteristics	Femoral Bifurcation (All) (n = 150)	REA + DCB (n = 66)	DCB Alone (n = 84)	*p*-Value REA + DCB vs. DCB Alone
Safety				
Minor complications (n, %)	2 (1%)	2 (3%)	0 (0%)	<0.05
Minor bleeding (n, %)	2 (1%)	2 (3%)	0 (0%)	0.048
Major complications (n, %)	4 (3%)	4 (3%)	0 (%)	<0.05
Embolism (n, %)	3 (2%)	3 (5%)	0 (0%)	<0.05
Major bleeding (n, %)	1 (0.7%)	1 (1.5%)	0 (0%)	0.269

**Table 4:** Three-year follow-up of PAD patients with femoral artery bifurcation stenosis following endovascular treatments. REA = rotational excisional atherectomy; DCB = paclitaxel-coated balloon. ABI = ankle brachialis index, cdTLR = clinical-driven target lesion revascularisation; MALEs = major adverse limb events; MACEs = major adverse cardiovascular events. Values are mean ± SD or are given as absolute values in %.

Follow-Up and Clinical Outcome Data	Femoral Bifurcation (All) (n = 150)	REA + DCB (n = 66)	DCB Alone (n = 84)	*p*-Value REA + DCB vs. DCB Alone
Patency n (%)				
One-year patency n (%)	128 (85%)	55 (83%)	73 (87%)	0.576
Three-year patency n (%)	125 (83%)	53 (80%)	72 (86%)	0.377
Secondary patency n (%)	146 (97%)	63 (95%)	81 (96%)	0.763
MALEs n (%)	28 (19%)	14 (21%)	14 (17%)	0.478
cdTLR (n, %)	25 (17%)	13 (20%)	12 (14%)	0.377
Major amputation n (%)	3 (2%)	2 (3%)	1 (1%)	0.493
Minor amputation n (%)	1 (1%)	0 (0%)	1 (1%)	0.869
All-cause mortality n (%)	15 (10%)	3 (5%)	12 (14%)	0.074
MACEs n (%)	12 (7%)	3 (5%)	9 (11%)	0.170
Sepsis death n (%)	3 (2%)	0 (0%)	3 (4%)	0.119
Wound healing (days)	107 ± 29	95 ± 26	120 ± 32	0.071
Ankle brachialis index				
ABI (treated leg)	0.76 ± 0.2	0.78 ± 0.1	0.76 ± 0.2	0.500
Rehospitalisation				
Three–year follow-up n (%)	61 (41%)	27 (41%)	36 (43%)	0.145
Cause for hospitalisation				
Cardiac	3 (2%)	1 (1.5%)	2 (2%)	0.616
Vascular	39 (26%)	18 (27%)	21 (25%)	0.616
Sepsis	8 (5%)	0 (0%)	8 (1%)	0.616
Vascular surgery	13 (9%)	8 (12%)	5 (6%)	0.616

**Table 5:** Predictors of (A) patency (B) cdTLR, (C) MALE-free survival, (D) MACE-free survival after EVT. REA = rotational excisional atherectomy, DCB = drug-coated balloon; BMI = body mass index; DM = diabetes mellitus; eGFR = estimated glomerular filtration rate, CLTI = chronic limb-threatening ischemia; IC = intermittent claudication; cdTLR = clinical-driven target lesion revascularisation; MALE = major adverse limb event; MACE = major adverse cardiovascular event.

**A. Patency**	**No. at Risk**	**Cumulative death n (%)**	**Hazard**		***p*** **-Value**
			Unadjusted	Adjusted (age, BMI, gender, DM, eGFR ≤ 60 mL/min; PAD stage, stent placement)	
REA + DCB	53	13 (20%)	0.9 (0.643–1.308)	0.9 (0.631–1.308)	0.608
				Age 1.0 (0.981–1.020)	0.993
				BMI 1.0 (0.957–1.048)	0.936
				Gender (male) 0.9 (0.645–1.420)	0.820
				DM 1.0 (0.693–1.403)	0.938
				eGFR ≤ 60 mL/min 1.0 (0.669–1.506)	0.985
				CLTI 0.9 (0.597–1.455)	0.757
				IC 1.1 (0.687–1.674)	0.757
				Stent placement 1.0 (0.682–1.385)	0.874
**B. cdTLR**	**No. at Risk**	**Cumulative death n (%)**	**Hazard**		***p*** **-Value**
			Unadjusted	Adjusted (age, BMI, gender, DM, eGFR ≤ 60 mL/min; PAD stage, stent placement)	
REA + DCB	52	13 (20%)	1.5 (0.622–3.482)	1.6 (0.615–4.237)	0.331
				Age 1.0 (0.981–1.020)	0.960
				BMI 1.0 (0.910–1.050)	0.933
				Gender (male) 0.9 (0.621–1.443)	0.798
				DM 1.0 (0.339–1.491)	0.932
				eGFR ≤ 60 mL/min 1.0 (0.685–1.510)	0.881
				CLTI 0.9 (0.534–1.545)	0.721
				IC 1.1 (0.689–1.677)	0.752
				Stent placement 1.0 (0.218–0.863)	0.925
**C. MALE**	**No. at Risk**	**Cumulative death n (%)**	**Hazard**		***p*** **-Value**
			Unadjusted	Adjusted (age, BMI, gender, DM, eGFR ≤ 60 mL/min; PAD stage, stent placement)	
REA + DCB	64	14 (21%)	1.3 (0.591–3.066)	1.4 (0.557–3.384)	0.492
				Age 1.0 (0.948–1.033)	0.628
				BMI 1.0 (0.953–1.124)	0.418
				Gender (male) 1.8 (0.794–3.914)	0.164
				DM 1.1 (0.426–2.729)	0.874
				eGFR ≤ 60 mL/min 1.0 (0.393–2.421)	0.957
				CLTI 1.8 (0.699–4.826)	0.217
				IC 3.9 (0.0.224–1.054)	0.080
				Stent placement 1.1 (0.525–2.371)	0.776
**D. MACEs**	**No. at Risk**	**Cumulative death n (%)**	**Hazard**		***p*** **-Value**
			Unadjusted	Adjusted (age, BMI, gender, DM, eGFR ≤ 60 mL/min; PAD stage, stent placement)	
REA + DCB	62	3 (5%)	0.4 (0.103–1.529)	0.3 (0.081–1.414)	0.137
				Age 1.0 (0.940–1.065)	0.984
				BMI 0.9 (0.783–1.021)	0.098
				Gender (male) 1.6 (0.447–5.787)	0.467
				DM 0.7 (0.153–3.328)	0.667
				eGFR ≤ 60 mL/min 1.0 (0.250–4.058)	0.992
				CLTI 5.0 (1.002–25.366)	0.050
				IC 4.1 (0.466–36.641)	0.074
				Stent placement 1.4 (0.427–4.807)	0.561

## Data Availability

Data are contained within the article and Supplementary Materials.

## Supplementary Materials

The following supporting information can be downloaded at: https://www.mdpi.com/article/10.3390/biomedicines12102213/s1, Table S1: Visual calcium scoring across different femoral segments according to duplex sonography. Score of 0 if no wall heterogeneity or anechoic shadowing was observed, a score of 1 if there was evidence of wall heterogeneity without anechoic shadowing, and a score of 2 if there was clear anechoic shadowing or high-grade stenosis or total occlusion.

## Abbreviations

ABI	ankle brachial index
ASA	acetylsalicylic acid
ALI	acute limb ischaemia
cdTLR	clinical-driven target lesion revascularisation
CFA	common femoral artery
CFEA	common femoral endarterectomy
CLTI	chronic limb-threatening ischemia
DCB	drug-coated balloon
EVT	endovascular treatment
MACE	major adverse cardiovascular events
MALE	major adverse limb events
NCDR	National Cardiovascular Data Registry
PFA	profunda femoris artery
POBA	plain old balloon angioplasty
PTA	percutaneous transluminal angioplasty
REA	rotational excisional atherectomy
SFA	superficial femoral artery
TEA	thromboendarterectomy
