# Supplementary material for: Three-Year Safety and Efficacy of Endovascular Treatment of Common Femoral Artery in 150 PAD Patients
Source: Biomedicines. 2024 Sep 27;12(10):2213. doi: 10.3390/biomedicines12102213 (PMC11505484; doi:10.3390/biomedicines12102213)
Supplement: Supplementary file 1 [file biomedicines-12-02213-s001.zip › biomedicines-3209929-supplementary.pdf]

Table S1: Visual calcium scoring across different femoral segments according to duplex sonography. Score of 0 if no wall heterogeneity or anechoic shadowing was observed, a score of 1 if there was evidence of wall heterogeneity without anechoic shadowing, and a score of 2 if there was clear anechoic shadowing or high-grade stenosis or total occlusion.

	Visual Calcium Scoring					
	REA+DCB (n=66)			DCB (n=84)		
	0	1	2	0	1	2
CFA	0	26	40	0	31	53
SFA	0	15	20	0	6	22
PFA	0	3	4	0	4	5

CFA = common femoral artery; SFA = superficial femoral artery, PFA = Profunda femoral artery.
